# Removal of heavy metals from industrial wastewater using microbial fuel cell

**DOI:** 10.1002/elsc.202200009

**Published:** 2022-08-03

**Authors:** Sameer Al‐Asheh, Marzieh Bagheri, Ahmad Aidan

**Affiliations:** ^1^ Department of Chemical Engineering American University of Sharjah Sharjah UAE

**Keywords:** heavy metals, MFC, precious metals, wastewater

## Abstract

Removal efficiency of gold from a solution of pure tetrachloroaurate ions was investigated using microbial fuel cell (MFC) technology. The effects of type of catholyte solution and initial gold concentration on the removal efficiency were considered. Due to its presence at high levels in the gold wastewater, the effect of copper ions on the removal efficiency of the gold ions was also studied. The effects of pH and initial biomass concentration on the gold removal efficiency was also determined. The results showed that after 5 h contact time, 95% of gold removal efficiency from a wastewater containing 250 ppm of initial gold ions at ambient temperature using 80 g/L yeast concentration was achieved. After 48 h of the cell's operation under the same condition, 98.86% of AuCl_4_^–^ ions were successfully removed from the solution. At initial gold concentration in the waste solution of 250 ppm, pH 2, and initial yeast concentration of 80 g/L, 100% removal efficiency of the gold was achieved. On the other hand, the most suitable condition for copper removal was found at a pH of 5.2, where 53% removal efficiency from the waste solution was accomplished.

## 1 INTRODUCTION

The growth in population and increase in demand lead to an increase in the number of industries, such as gold mining, petroleum refinery, mining, textile, and batteries [1]. This has also resulted in an increase in the amounts of effluents that must be discharged from these industries into the environment. These effluents may contain significant proportions of contaminants, such as heavy metals [2], which can easily be absorbed by living organisms. Their introduction into the food chain may result in accumulation in large quantities in human bodies. Due to their harmful effects, heavy metals have maximum allowable limits in human body, which if exceeded will result in severe disorder and diseases [3]. Hence, it is of utmost importance that the effluents from such industries are treated before being discharged into the environment.

There are several methods that can be used to treat industrial effluents containing heavy metals, such as solvent extraction, filtration, ion exchange, coagulation, sedimentation, oxidation, and adsorption. However, these techniques have several disadvantages; for example, high cost, low removal efficiency, regeneration, and the problem of secondary contaminations [4]. Therefore, it is proposed to implement new techniques which are more cost effective, have a higher removal efficiency and have less susceptibility to secondary contamination. Among these methods is the use of microbial fuel cell (MFC) technology.

MFC is a technology developed to convert the stored energy in the chemical compound to electrical energy by using microorganisms. Basically, the microorganisms degrade the organic matter while simultaneously producing energy. Electricity generation is one of the most important outcomes of the MFCs which results in tremendous usage in different applications such as spacecraft and systems that require only low power to transmit signals. Additionally, hydrogen production is another application of the MFC which requires applying external power. Because the generation of hydrogen from protons and produced electrons by metabolic reaction of microorganisms is thermodynamically unfavorable, an external potential is applied to increase the cathode potential and allow the reaction to become favorable [5]. Finally, yet importantly, another application of MFCs is to treat wastewater and industrial effluents [6]. The extraction of energy from various waste and converting it to electricity using MFC has been recently reviewed by Elhenawy et al. [7]. Other applications include used of MFC in water desalination [8] and in sensors and biosensors [9].

**FIGURE 1:**
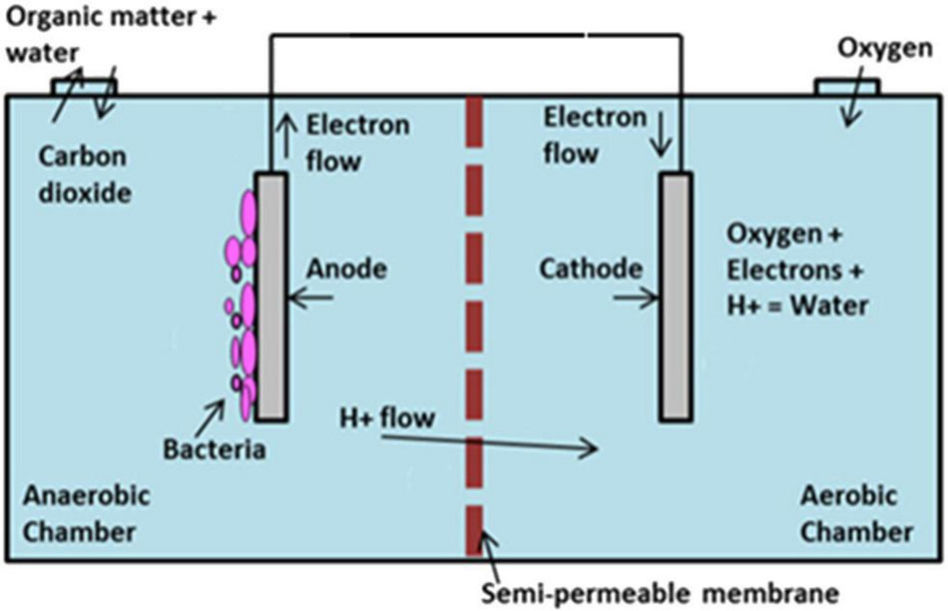
Schematic representation of MFC [12]

The working principle of MFC is shown in Figure 1. The MFC consists of two chambers, that is, anodic and cathodic chambers. Each chamber can be made of glass, polycarbonate, or Plexiglas, and each contains an electrode which can be carbon paper, carbon‐cloth, graphite, graphite felt, Pt, Pt black, or reticulated vitreous carbon. The chambers are divided using a proton exchange membrane (PEM) [10]. Protons and electrons are produced by the microorganisms in the anodic chamber due to decomposing the organic matter; the electron transfers to the cathodic chamber from the anodic electrode to the cathodic electrode using external electrical circuit, while the proton transfers through the PEM membrane to the cathodic chamber. From the thermodynamic viewpoint, the anode compartment should always have higher potential than the cathode compartment. Thus, allowing the electrons to spontaneously transfer from the anode to the cathode chamber without any requirement for external power. The cathodic chamber has a high potential electron acceptor where reduction reaction takes place in this chamber. The electron acceptor should ideally be nontoxic and not interfere with the microbes. An example of such an electron acceptor is oxygen, which is nontoxic and easily available. Additionally, besides oxygen, ferricyanide and heavy metals can also be used as an alternative electron acceptor [11].

**PRACTICAL APPLICATION**

Heavy metals are potential pollutants that have to be removed from wastewater effluents. Gold industry effluent is used as a model of wastewater effluent. One of the applications of microbial fuel cell (MFC) is to use it as a technique for removal of heavy metals, which of the subject of this paper. The MFC unit was devised for this purpose and effect of existence of other metals on this removal process is considered.

Accordingly, MFC can be defined as a device which converts chemical energy into electrical energy through the use of microbe as catalyst [13]. The microbe presents as a biofilm on the surface of the anode in the MFC and acts as a biocatalyst employed to carry out the electrochemical redox reactions [14]. The types of redox reactions that can take place in the MFC are greatly dependent on the type of organic matter and the electron acceptor used in the MFC. Examples of the possible oxidation/reduction reactions for different substrates are displayed in Table 1.

**TABLE 1:** Possible oxidation and reduction reactions [15]

Oxidation reactions at the anode		
Substrate	Reaction	E (V)
Acetate	$CH_{3}\mathrm{CO}O^{-}+3H_{2}O\to CO_{2}+\mathrm{HC}O_{3}^{-}+8H^{+}+8e^{-}$	–0.3
Glucose	$C_{6}H_{12}O_{6}+6H_{2}O\to 6CO_{2}+24H^{+}+24e^{-}$	–0.429
Glycerol	$C_{3}H_{8}O_{3}+6H_{2}O\to 3\mathrm{HC}O_{3}^{-}+17H^{+}+14e^{-}$	–0.289
Domestic wastewater	$C_{10}H_{19}O_{3}N+18H_{2}O\to 9CO_{2}+NH_{4}^{+}+\mathrm{HC}O_{3}^{-}+50H^{+}+50e^{-}$	——‐

Reduction reaction at the cathode		
Substrate	Reaction	E (V)
Oxygen	$O_{2}+4H^{+}+4e^{-}\to 2H_{2}O$	1.23
	$O_{2}+2H^{+}+2e^{-}\to H_{2}O_{2}$	0.27
Nitrate	$NO_{3}^{-}+2e^{-}+2H^{+}\to NO_{2}^{-}+H_{2}O$	0.43
	$2NO_{3}^{-}+10e^{-}+12H^{+}\to N_{2}+6H_{2}O$	0.73
Ferric ion	$Fe^{3+}+e^{-}+H^{+}\to Fe^{2+}+1/2\;H_{2}O$	0.77

Microbial fuel cells can be used to produce energy while treating a wastewater containing heavy metals to decrease their concentrations to the allowable levels before discharge into the environment. Metal pollutants, such as chromium, copper, vanadium and mercury, have been removed using two chambered MFC cells [16,^17^]. Heavy metals in MFCs are removed through the reduction of the cathode metal in the anaerobic cathodic chamber, while in the anodic chamber, organic matters are used as sources of carbon and electron donors [18]. It has been demonstrated that such processes as biosorption and precipitation reactions (i.e., sulfides and hydroxides) greatly aided in the removal of heavy metals from wastewater in the MFC system [18]. Table 2 presents a summary of previous studies on the removal of different metals using MFC technologies along with the maximum removal and maximum power generation.

**TABLE 2:** Summary of heavy metals removal using MFCs

Metal	MFC fabrication	Maximum removal recovery	Maximum power generation	Substrate	References
Cu (II)	Single‐chamber MFC, Carbon brush for anode, Carbon cloth/Pt coated for cathode	98.3%	10.2 W/m^3^	Sludge	[19, 20]
Cu (II)	Two‐chamber MFC, Graphite plate for anode, Graphite foil for cathode	99.88% (Anaerobic) 99.95 % (Aerobic)	0.43 W/m^2^ (Anaerobic) 0.80 W/m^2^ (Aerobic)	Acetate	[21]
Cr (VI)	Two‐chamber MFC, Carbon felt for anode, Carbon cloth(a)/Carbon brush(b)/Carbon felt(c) for cathode	100% (a) 33.45% (b) 12.72% (c)	1221.91 mW/m^2^	Acetate	[22]
Cd (II)	Single‐chamber MFC; Carbon cloth for anode; Carbon cloth/Pt coated for cathode	90%	3.6 W/m^2^	Sewage sludge	[22]
Hg (II)	Two‐chamber MFC, Graphite felt for anode, Carbon paper for cathode	99.54% (for 100 mg/L Hg (II))	433.1 mW/m^2^	Mixture of sludge with artificial wastewater	[23]
Ag (I)	Two‐chamber MFC, Carbon brush for anode, Carbon cloth for cathode	99.91% (for 50 ppm Ag (I))	109 mW/m^2^	Mixture of sludge with artificial wastewater	[24]
Au(III)	Two‐chamber MFC Carbon brush for anode, Carbon cloth for cathode	99.88% (for 200 mg/L Au(III))	6.58 W/m2	Mixture of sludge with artificial wastewater	[25]

This work aims to study the possibility of recovering precious metals such as gold from metal containing solutions using MFC. Removal of gold using MFC has been considered before, therefore, it is expected that the outcomes of this work will have an add value to this area of research. The effect of presence of other metals, namely copper, in the solution on the removal of gold using MFC is considered in this work. The study also investigates the effect of different parameters on the performance of MFC's, such as initial gold concentration, initial pH, initial yeast concentration, and type of catholyte solution, on the gold removal efficiency. The removal efficiency of Au(III) ions from real industrial wastewater, where other heavy metals such as copper, chromium, lead etc. may exist, is also considered.

## 2 MATERIALS AND METHODS

### 2.1 Materials

The following materials have been utilized in this work:

i. Buffer solution: 0.1 M potassium phosphate buffer prepared by mixing 61.5 mL of potassium hydrogen phosphate (1 M) with 38.5 mL of potassium dihydrogen phosphate (1 M) while making the solution up to 1 L with deionized water [19].
ii. Biomass: different concentrations of biomass, namely 50, 80, 100 g/L, were prepared by activation of dried yeast powder using glucose as nutrient. The yeast powder was purchased form a commercial market and it was manufactured by DCL.
iii. Mediator solution: 0.01 M methylene blue (MB) prepared by dissolving 1.87 g of MB powder in a 500 mL buffer solution. The prepared mediator solution was stored at room temperature for further use.
iv. Catholyte solutions: different types of catholytes were used to study the effect of existence of other types of heavy metals on the efficiency of the gold removal and recovery processes. These namely represent: a 50 mL solution with a concentration of 500 ppm tetrachloroaurate using deionized water; 50 mL buffered solutions of tetrachloroaurate ions with initial concentrations of 500 ppm, 250 ppm, and 125 ppm; 50 mL solution containing buffered mixture of tetrachloroaurate and copper ions using with initial concentrations of 250 ppm and 1000 ppm of Au(III) and copper of, respectively; 50 mL wastewater samples collected from effluent of one of the gold refineries in the UAE with initial concentrations of Au(III) and copper were 250 ppm and 1300 ppm, respectively.
v. Anolyte solution (microbial medium): 25 mL solution of a mixture of biomass, methylene blue as mediator, and buffer solution to control the pH.
vi. Anode and cathode electrodes: carbon cloth and carbon brushes were used as cathode and anode, respectively. The carbon brushes were hand made using strips of carbon cloth braided on the steel wire.
vii. Membrane: Nafion membrane is used to separate the cathodic compartment from anodic compartment while allowing only proton diffusion between the two compartments.
viii. MFC: the cell was constructed from rectangular blocks made of acrylic. To ensure no leakage present, rubber gaskets were used between the rectangular blocks. The cell was held together using clamps.

### 2.2 Instrumentations

Different types of instruments were used to control and examine the operating conditions for the investigation of gold and copper removal and performance of the MFC. pH was measured using via HANNA HI2020 benchtop pH meter. VARIAN Atomic Absorption Spectroscopy (AAS) was used to measure gold and copper concentration with proper lamps. Open circuit potential was evaluated by using digital FLUKE 87‐V‐Eur industrial multimeter. A small magnetic chip is placed inside the anodic chamber to mix the microbial medium.

### 2.3 Experimental setup

In this study, MFC unit made from the acrylic rectangular blocks was implemented. It is composed of two chambers, the first chamber is the anodic chamber containing the microbial medium and the anode where continuous mixing is required as mentioned in previous studies [26]. A small magnetic chip is placed inside the anode chamber while the whole cell is placed on the magnetic stirrer base to ensure proper mixing. Carbon brush is used as an electrode due to its higher surface area compared to carbon cloth electrodes. The anionic chamber is split by the Nafion membrane while placing a gasket rubber sheet in both sides to prevent leakage. Pretreatment of the membrane was performed by keeping it initially for 1 h in boiling deionized water and then soaking in deionized water at room temperature for 24 h before its usage inside the cell to allow hydration and expansion of the pores [27]. The second chamber, referred as heavy metals removal chamber, where the desired catholyte solutions are fed. The carbon cloth in this case is utilized as an electrode. The cell is operating under anaerobic conditions. Therefore, all chambers were purged with nitrogen to remove dissolved oxygen inside each chamber and then the holes on top of each chamber were sealed. The cathodic chamber held double the volume of the anodic chamber (50 mL in cathodic chamber and 25 mL in anodic chamber) [28].

### 2.4 Biomass (yeas) activation

Different biomass concentrations (50, 80, 100 g/L) were used in this study. To prepare a biomass of 100 g/L, for example, 2.5 g of DCL activated dried yeast was added to 25 mL of water at a temperature of 38°C, followed by addition of 3.75 g of glucose to the mixture as a nutrient for the yeast. Next, the top of beaker was covered with plastic cling foil while covering the whole beaker with a large cloth to allow resting and warm environment for yeast activation. The solution was left for around 20 min until a layer of sludge with bubbles was created on the top of the medium. At this stage, it is assumed that the yeast has been activated properly.

### 2.5 Experimental procedure

The performance of MFC toward removing pure tetrachloroaurate ions is investigated using different catholyte solutions. After determining the proper catholyte solution, the effect of different initial Au(III) on removal efficiency was considered. Two different catholytes were used; one using Au(III) with a buffer solution, while the other solution was made without buffer using distilled water, both having an initial concentration of 500 ppm Au(III) and total volume of 50 mL. After pretreatment of the membrane and purging the whole cell with nitrogen, the MFC was ready to operate. The voltmeter was connected to the electrodes before filling the compartments with their respective solutions and starting the cell. After successful installation, that is, testing leakage, and operating the cell, 0.1 mL samples of catholyte solutions were withdrawn periodically to analyze the gold concentration with time. This amount of sample volume does not affect significantly the volume of the cathode chamber and its operating conditions. Samples from the catholyte were taken every 5 min, 15 min, 25 min, 1 h, 2 h, 3 h, 4 h, 5 h, 24 h, and 48 h after starting the cell. The pH, temperature, and the OCP (open circuit potential) were also recorded at each of these periods. After 24 h of operation, a mixture of 1 mL yeast, 0.4 mL methylene blue, and 3 mL buffer was added to the anolyte solution to prevent any reduction in the number of yeast cells.

The removal efficiency of the metal (either gold or copper) was calculated using the following expression:

(1) $$\text{Metal}\;\text{removal}\;\text{efficiency}\;\left(\%\right)=\frac{C_{0}-C_{t}}{C_{0}}*100$$

where the *C*_0_ represents the initial gold concentration inside the catholyte (ppm) and $C_{t}$ is the gold concentration inside the catholyte at time *t*.

Another important parameter that was evaluated is the efficiency of gold recovery. It was calculated using the mass deposited on the electrode surface (*M_d_*) divided by the mass removed from the catholyte:

(2) $$\begin{array}{c} \text{Gold}\;\text{recovery}\;\text{efficiency}\;\left(\%\right)=\frac{\mathrm{Md}}{\left(C_{0}-C_{t}\right)*V_{\mathrm{cat}}}*100 \end{array}$$

where $V_{\mathrm{cat}}$ is the volume of cathode chamber (L). The mass deposited on the electrode surface was determined by brushing the electrode surface.

The possible reactions that can take place are:

i. Anodic compartment
(3) $$\begin{array}{ccc} & & C_{6}H_{12}O_{6}+6H_{2}O\to 6CO_{2}+24H^{+}+24e^{-};\\ & & \;E^{0}=\;-0.429\;V \end{array}$$
ii. Cathodic compartment
(4) $$\mathrm{AuC}l_{4}^{-}+3\;e^{-}\to \mathrm{Au}\left(s\right)+4Cl^{-};\;\;E^{0}=\;0.994\;V$$
(5) $$2H^{+}+2e^{-}\to H_{2};E^{0}=0V$$
(6) $$2Cl^{-}\to Cl_{2}+2e^{-};\;\;E^{0}=\;-1.35\;V\;$$
(7) $$2\mathrm{AuC}l_{4}^{-}+3H_{2}2\mathrm{Au}\left(s\right)+8Cl^{-}+6H^{+}$$
(8) $$H_{2}+Cl_{2}\to 2\mathrm{HCl}$$

The operation was repeated but at different initial concentrations of Au(III). It was also repeated with different catholyte solution, where 50 mL of pure copper ions in the buffer having initial concentration of 1000 ppm were fed to the cathode chamber. In addition, a 50 mL solution of 250 ppm Au(III) was mixed with 1000 ppm Cu^2+^ was also fed to the cathode chamber in a separate operation. In addition to the above‐mentioned reactions in the cathode chamber, the following reactions expect to take place in the presence of copper:

(9) $$Cu^{2+}+2e^{-}\to \mathrm{Cu}\left(s\right);E^{0}=0.337V$$

(10) $$\begin{array}{c} 2Cu^{2+}+H_{2}O+2e^{-}\to Cu_{2}O+2H^{+};\;\;E^{0}=\;0.207\;V \end{array}$$

(11) $$\begin{array}{c} Cu_{2}O+2e^{-}+2H^{+}\to 2\mathrm{Cu}\left(s\right)+H_{2}O;\;\;E^{0}=\;0.059\;V \end{array}$$

The removal efficiency of Au(III) from actual industrial wastewater was also evaluated, where 50 mL of wastewater was fed to the cell as catholyte. The sample provided by gold refinery was collected from the post‐treatment with concentration of gold ions being less than 1 ppm. Thus, Au(III) was added to the solution to increase the gold concentration to 250 ppm. This concentration was chosen to ensure proper operation of the cell and to ensure consistency with the pre‐treatment. The actual pH of the raw catholyte solution form the waste effluent was 0.2. Solutions at different pHs, namely, 2, 2.65, 4.45, and 5.2, were prepared to investigate the effect of pH. Finally, different initial biomass concentrations, namely 50 g/L, 80 g/L, and 100 g/L, were used to evaluate their influence on the removal efficiency of both gold and copper ions from the wastewater solution. The experiments were repeated twice, and average values are reported.

## 3 RESULTS AND DISCUSSION

### 3.1 Removal of pure tetrachloroaurate ions from aqueous solutions

#### 3.1.1 Effect of catholyte

**FIGURE 2:**
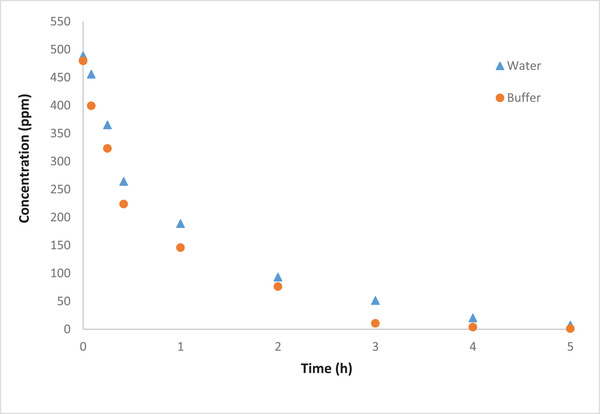
Concentration of [AuCl^–^_4_] during MFC operation using unbuffered and buffer as catholyte solutions

Two different types of catholytes, water and buffer, were used to determine the gold removal efficiency and consequently determine the most suitable catholyte solution. The cell was operated for 48 h with an initial Au(III) concertation in both catholyte solutions of 500 ppm. The results, Figure 2, show that rate of Au removal is slightly faster in buffer solution than that of unbuffered aqueous solution. This is expected as buffer acts as a catholyte solution and thus the conductivity increased due to the increase in the number of ions (i.e., combination of buffer ions and gold ions). However, changing the catholyte solution does not impact the removal efficiency significantly, 99.9% removal for both cases. On the other hand, 83.09% of Au was recovered by brushing the electrode surface when the buffer solution was used as catholyte, while for the water solution the recovery rate was found to be 80.78%.

**FIGURE 3:**
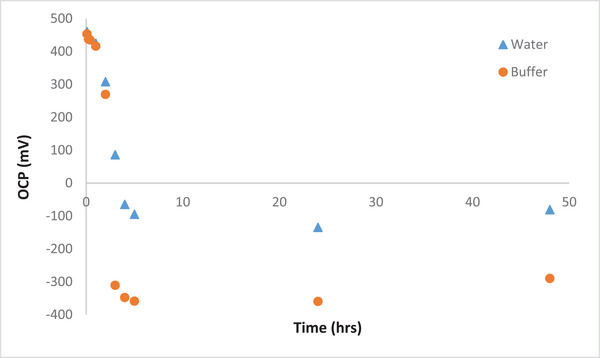
Open circuit potential using buffer and water as catholyte solutions with 500 ppm AuCl^–^_4_ initial concentration

Figure 3 shows the open circuit potential (OCP) of the cell during the operation of the cell over 2 days. It is seen that after approximately 6 h of operation, the OCP values became negative. In general, a negative value of OCP could be attributed to two factors:

i. The lack of biomass concentration which results in limited electron production. The occurrence of this problem was circumvented by using an appropriate initial concentration of the yeast and refilling of the anolyte solution after 24 h of the cell operation with fresh active yeast. The existence of bubbles in the anode compartment is an indication of the proper performance of the microorganisms. Thus, this cannot be the reason behind the negative OCP values.
ii. The negative values of OCP can be assumed because of the reduction reaction of $Cl^{-}$ ions. After 6 h of cell operation, significant reduction in gold concentration occurred, and thus considerable amounts of $Cl^{-}$ were created due to the reaction in the cathodic chamber (as shown above). However, in this case since the potential of this reaction is less than the anodic reaction, the electrons will flow from the cathode to the anode chamber which results in the negative OCP. It is worth mentioning that the remaining electrons inside the cathode chamber will be utilized for further reduction of Au(III).

**FIGURE 4:**
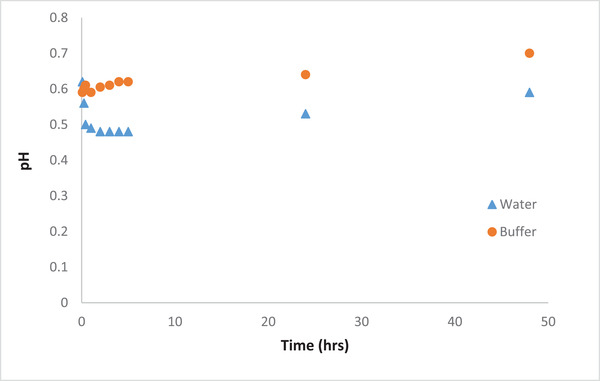
Variation of pH during MFC operation using water and buffer solutions at 500 ppm initial AuCl^–^_4_ concentration

The pH was also recorded during the operation of the cell. Since the hydrogen evolution reaction is thermodynamically favorable, the hydrogen gas and chlorine gas (H_2_ and Cl_2_) may also be generated, reactions (3) and (4). The existence of these gases was noticed as evidenced by the presence of bubbles in the cathode compartment during the operation of the cell. It can be said that the use of buffer as catholyte solution can increase the removal efficiency by neutralizing the H^+^ ions. Consequently, the reduction reaction of protons is diminished which results in higher and faster removal efficiency of Au ions as discussed earlier. This is also demonstrated by the limited fluctuation in the pH in the case of using buffer solution compared to that of water solution (Figure 4). Therefore, the buffer solution was utilized as catholyte for the further tests.

#### 3.1.2 Effect of initial gold concentration

The effect of initial gold concentration on the performance of the cell was investigated using initial concentrations of 125, 250, 500 ppm. The removal efficiency as well as recovery efficiency of each run are presented in Table 3.

**TABLE 3:** Gold ions removal and recovery efficiency at different initial gold concentrations

	[Au] = 500 ppm	[Au] = 250 ppm	[Au] = 125 ppm
Removal %	99.90	98.30	95.52
Recovery %	83.10	81.05	83.30

**FIGURE 5:**
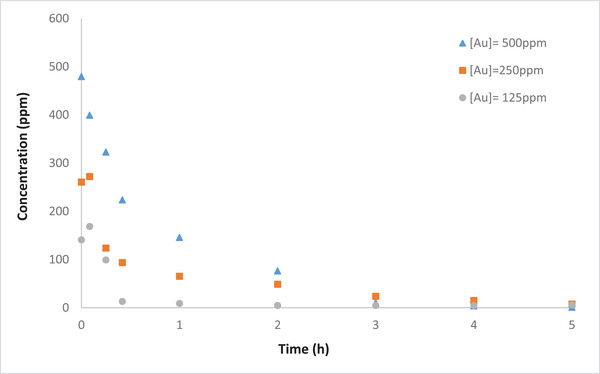
Effect of initial gold concentration on its removal during MFC operating

Figure 5 shows the removal efficiency of each run during the operation. As the gold concentration increased the removal efficiency, as well as OCP, increased. This is expected due to the increase in conductivity and equilibrium potential, based on the Nernst equation. Although the highest removal efficiency was achieved with higher concentration of Au(III) (99.9% and 98.3% for initial gold concentration of 500 ppm and 250 ppm, respectively), 250 ppm was chosen for further experiments.

### 3.2 Removal of gold ions in presence of copper

**FIGURE 6:**
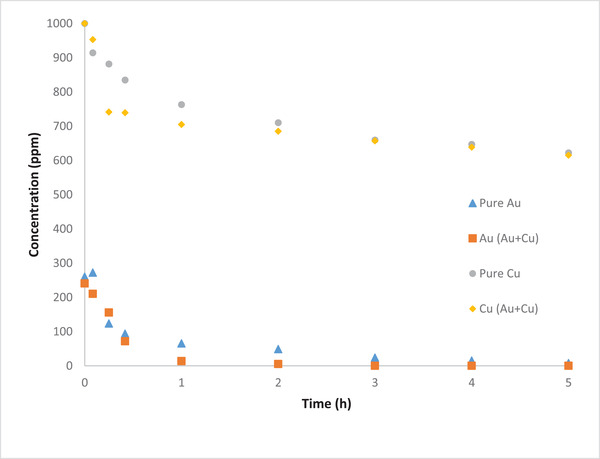
Variation of Au and Cu during MFC operation for different catholyte solutions

Since the effluent of gold refineries and mining has a high content of copper ions [29], investigation of the effect of copper on the removal efficiency of gold ions is of utmost importance. A mixture of 250 ppm and 1000 ppm of gold and copper ions, respectively, was prepared using buffer solution. As previously, the cell was operated for 48 h. Figure 6 shows the variations in the concentration of both Cu and Au during MFC operation for the different scenarios. It is worth noting that in such case where other metal present, the calculation of recovery efficiency is not possible since other heavy metals will deposit with the gold atoms on the cathode surface. According to Zhang et al. [30], the Cu atoms formed on the electrode surface after the Au atoms and that different metals deposit in distinct layers on the surface. They also reported a 95.4% gold removal, when the gold and copper ions coexisted. In this work, the gold removal efficiency was found to be 99.81% after 48 h, which is higher than the one obtained from solutions containing only gold ions. The reason behind this could be due to the increase in the number of ions (Cu and Au) and thus an increase in the conductivity.

**FIGURE 7:**
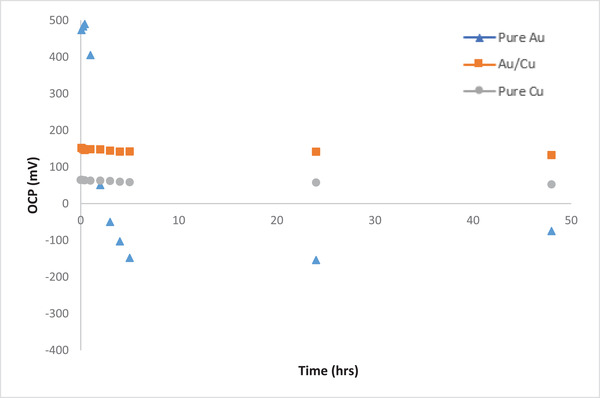
Variation of OCP (mV) during MFC operation for different catholyte solutions

The OCP plots for pure Au, pure Cu and mixture of Au/Cu are also shown in Figure 7. It is seen that for solution of Cu ions only, negative OCP cannot be observed since Cl^–^ ions cannot be generated in this case. When Cu ions coexist with Au ions, still negative OCP is not observed, but significant reduction in the OCP is noticed. It could be possible that the reaction mentioned above in the cathodic chamber for Cl_2_ is not favorable compared to other reactions.

### 3.3 Removal of gold ions from industrial wastewater

**FIGURE 8:**
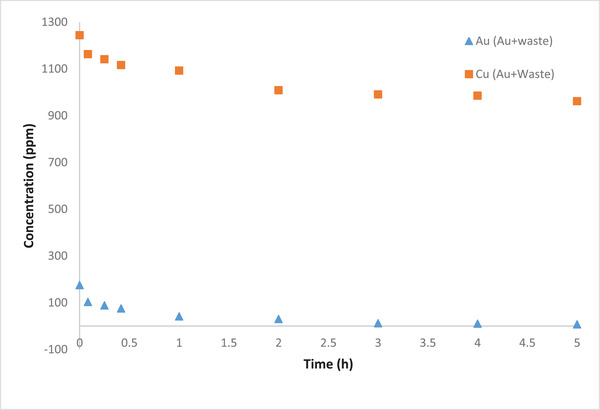
Concentration profiles of Au and Cu using industrial waste solution

Removal of gold ions in the presence of other heavy metals in the industrial gold refinery waste was investigated. To the best of the authors’ knowledge, such study has not been considered before. The variations of Au and Cu concentrations during the MFC operation are shown in Figure 8. The results indicate that the removal efficiency of gold ions is slightly increased compared to the case when Cu present with Au; from 98.86% to 99.81%. The low removal of Cu could be attributed to the presence of other heavy metals in the wastewater such zinc, lead, etc. [31] which may compete/prevent Cu reduction reaction in the cathodic chamber. Hence, it is possible that the reduction reactions of other heavy metals in the cathodic chamber are more favorable to take place.

#### 3.3.1 Effect of pH

pH can influence the removal efficiency of Au in two aspects. Proton involvement is one of the aspects, however, since the reduction of gold ions protons is not involved, the effect of pH due to proton involvement on the removal efficiency is negligible. The other aspect is the stability of $\mathrm{AuC}l_{4}^{-}$ ions. According to the predominance diagram of Au(III)−OH−−−Cl− species [33], there are two factors that can affect the solubility of the $\mathrm{AuC}l_{4}^{-}$ ions in the solution; pH and the $Cl^{-}$ concentration. These two factors should be controlled to keep the $\mathrm{AuC}l_{4}^{-}$ ions soluble. If these two factors are not controlled appropriately, the $\mathrm{AuC}l_{4}^{-}$ ions will precipitate as Au(OH_3_). As the pH gets higher, the lower limit of $Cl^{-}$ concentration will increase in order to keep the $\mathrm{AuC}l_{4}^{-}$ ions stable in the solution. In this study, although all the samples were prepared with 250 ppm initial gold concentrations; the initial gold concentration was lower in the pH range of 4.46–5. It appears that the concentration of the $Cl^{-}$ ions was not high enough to keep the $\mathrm{AuC}l_{4}^{-}$ ion stable within this range of pH. A dark yellow color precipitate was also observed at the end of the experiments which can be reasonably assumed to be a precipitate of Au(OH_3_).

The results (Table 4) revealed highest and fastest gold removal efficiency at pH 2. As the pH decreases, the rate of proton diffusion from cathode chamber to the anode chamber increases. This increase in diffusion rate has a negative impact on the activity of the microorganisms. Hence, the electron production decreases as the microorganisms are being negatively impacted.

**TABLE 4:** Removal of Au and Cu from industrial gold solution at diffent pH

Au/waste	pH 0.2	pH 2	pH 2.65	pH 4.45	pH 5.2
Au‐ Removal %	98.86	100.00	99.55	95.63	81.51
Cu‐ Removal %	31.60	34.06	34.48	44.44	52.98

For the pH range of 2–4.46, according to the predominance diagram [25], as mentioned earlier, the initial concentration of the Au ions is dropped mainly due to the precipitation. In addition, it seems that the reduction reaction of Au ions takes place first as long as a significant amount of gold ions are removed. After this step, the reduction reaction of other heavy metals is initiated. This hypothesis is supported by the low removal efficiency of the copper ions within 48 h of the cell operation as well as OCP reduction within this pH range.

**FIGURE 9:**
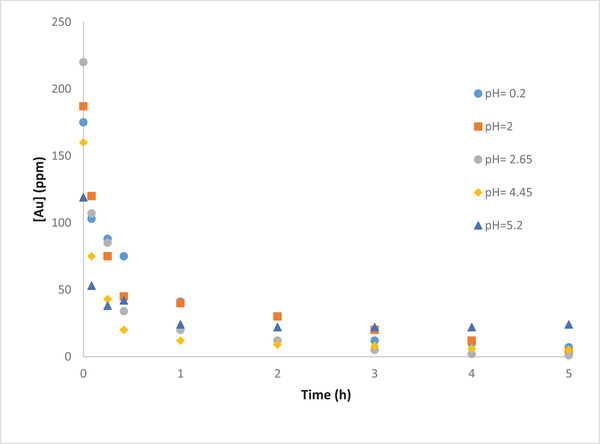
Effect of initial pH on removal efficiency of gold during MFC operation using gold waste solution

**FIGURE 10:**
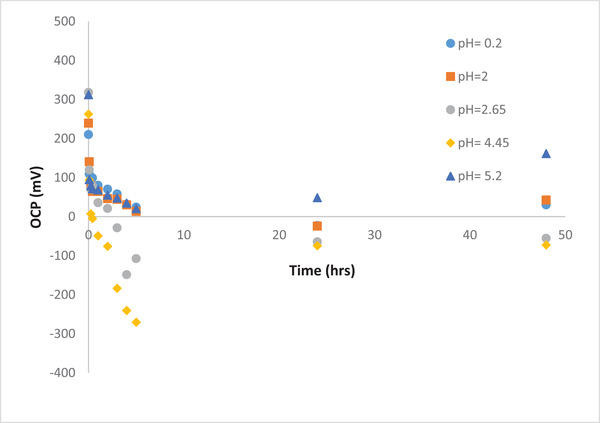
Effect of pH on OCP (mV) during MFC operation using gold waste solution

As the pH increases from 4.45 till 5, more gold ions precipitate. Hence, the initial concentration of gold is even less than before (i.e., 119 ppm), as illustrated in Figure 9. Nevertheless, it seems that in this case the reduction reaction of other heavy metals is taking place along the reduction reaction of gold ions. This is the reason behind the higher OCP values as well as higher Cu removal efficiency at this specific pH range (Figure 10).

For the effect of pH on copper removal, it seems pH does not influence the reduction reaction of Cu^2+^ to Cu(s), reaction (7). This reaction is solely dependent on the concentration of Cu^2+^. However, the reduction reaction of Cu^2+^ to Cu_2_O is a function of not only the concentration of Cu^2+^ but also it is pH dependence. Thus, increasing the pH causes the reaction in Equation (8) to take place more. Moreover, the reduction of Cu_2_O to Cu(s) depends only on the pH and according to Equation (9) it is favored within the lower pH ranges.

According to the Nernst equation, the equilibrium potential of the cathode for the different reactions at different pHs and Cu^2+^ concentrations can be calculated, and thus the most favorable reaction can be determined at given conditions. The Nernst equations for the three reactions are shown below:

(12) $$\begin{array}{c} E_{\mathrm{cat}}\;\left(Cu^{2+}/\mathrm{Cu}\;\right)=E_{\mathrm{cat}}^{0}\;\left(Cu^{2+}/\mathrm{Cu}\;\right)-\frac{\mathrm{RT}}{\mathrm{nF}}\ln\left(\frac{1}{\left[Cu^{2+}\right]}\right) \end{array}$$

(13) $$\begin{array}{c} \begin{array}{c} E_{\mathrm{cat}}\;\left(Cu^{2+}/Cu_{2}O\;\right)=E_{\mathrm{cat}}^{0}\;\left(Cu^{2+}/Cu_{2}O\;\right)-\frac{\mathrm{RT}}{\mathrm{nF}}\ln\left(\frac{{\left[H^{+}\right]}^{2}}{{\left[Cu^{2+}\right]}^{2}}\right) \end{array} \end{array}$$

(14) $$\begin{array}{c} E_{\mathrm{cat}}\;\left(Cu_{2}O/\mathrm{Cu}\;\right)=E_{\mathrm{cat}}^{0}\;\left(Cu_{2}O/\mathrm{Cu}\;\right)-\frac{\mathrm{RT}}{\mathrm{nF}}\ln\left(\frac{1}{{\left[H^{+}\right]}^{2}}\right) \end{array}$$

**FIGURE 11:**
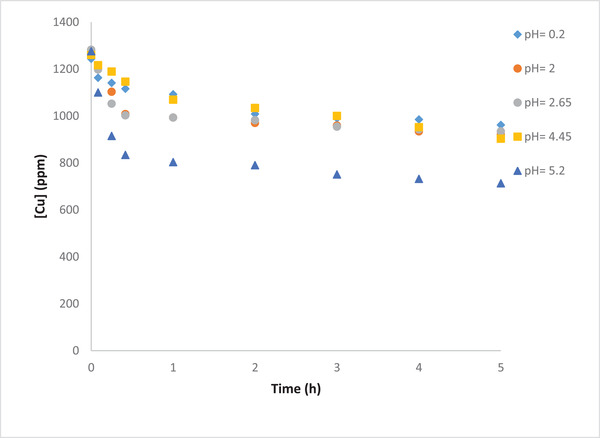
Effect of initial pH on Cu removal from the gold waste solution

Based on copper speciation with pH [33], for the 0.02 M initial Cu^2+^ concentration in the waste samples, as the pH increases from 2 to 5.2, the potential for the Cu^2+^ to Cu_2_O increases from 0.155 to 0.395 V; keeping in mind that the potential for the Cu^2+^ to Cu(s) reaction is independent of the pH. At 0.02 M Cu^2+^ concentration, the cathode potential is 0.285 V. As a result, for pH levels above 2.65, the reduction reaction of Cu^2+^ to Cu_2_O becomes more favorable. These observations indicate that increasing the pH would increase copper removal. This is consistent with the results obtained in this work as shown in Table 4 and Figure 11. It is seen that removal efficiency of the Cu^2+^ increases with the increase in its initial concentration, and that pH 5.2 is the most suitable condition for copper removal. This suggests that the removal efficiency of the copper ions is more favorable at a relatively more basic media.

#### 3.3.2 Effect of initial yeast concentration

**FIGURE 12:**
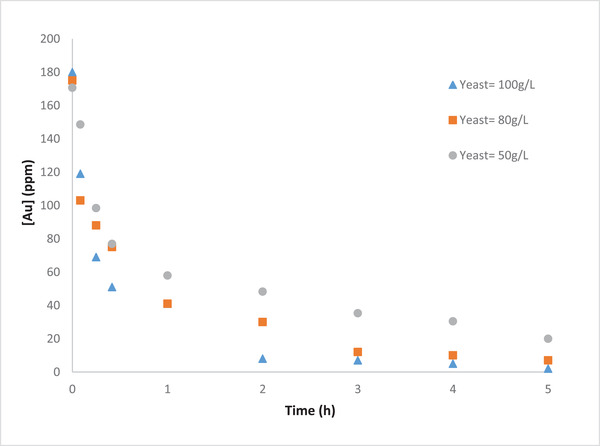
Effect of initial yeast concentration on removal of Au from the gold waste solution

**FIGURE 13:**
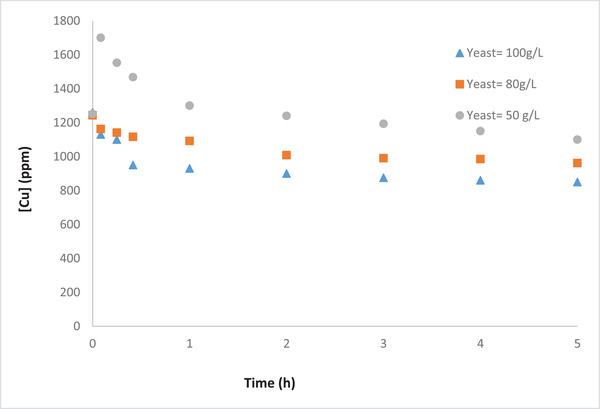
Effect of initial yeast concentration on removal of Cu from the gold waste solution

To explore the effect of initial yeast concentration on the gold removal efficiency, different concentrations of yeast, namely 50, 80, 100 g/L, were implemented. The results (Figures 12 and 13) showed that the initial yeast concentration has a positive effect on both gold removal efficiency. The increase in initial yeast concentration results in a further increase in removal of both Au and Cu from the gold waste solution. This is an expected results due to the increase in the release of electrons in the anodic chamber and thus enhancement of the reactions in the cathodic chamber. These results were also supported by the higher reduction in the OCP with the increase in yeast concentration (data not shown).

## 4 CONCLUSION REMARKS

MFC technology can be used to remove gold ions from the wastewater in the presence of other metal ions. The existence of other heavy metals does not hinder the operation of MFC for removing gold ions. It is thus a promising technology for heavy metals removal. The results revealed more than 95% removal of gold achieved after 5 h of cell's operation. This amount increased to 98.86% after 48 h of operation for a waste sample having 250 ppm initial gold concentration. The effects of different parameters (pH, initial yeast concentration, initial gold concentration, and type of catholyte) on the performance of the MFC were assessed. The initial yeast concentration of 80 g/L at the pH 2 under room temperature is the most optimum condition for removal of gold metals from a real waste solution; at such condition, 100% removal efficiency has been achieved. The MFC can be used to remove heavy metals from industrial wastewater effluence.

## CONFLICT OF INTEREST

The authors declare that they have no known competing financial interests or personal relationships that could have appeared to influence the work reported in this paper.

## Data Availability

The data that support the findings of this study are available from the corresponding author upon reasonable request.
